# Effectiveness of Hand Hygiene Interventions in Healthcare Facilities in Low- and Middle-Income Countries (LMICs): A Systematic Review

**DOI:** 10.64898/2026.07.28.26359185

**Published:** 2026-07-31

**Authors:** Fred Tusabe, Ryan Cronk, Fred Twinomugisha, Mackline Ninsiima, Darcy Anderson, Lucy K Tantum, Kenneth Kobba, Reuben Kiggundu, Dathan Byonanebye, Judith Nanyondo Semanda, Francis Kakooza

## Abstract

**Background:**

Hand hygiene is a core component of infection prevention and control, yet evidence on the effectiveness of hand hygiene interventions in health-care facilities in low-income and middle-income countries has not been comprehensively synthesized alongside implementation barriers and enablers. We assessed the effectiveness of hand hygiene interventions in these settings and examined implementation conditions shaping success.

**Methods:**

We conducted a systematic review drawing on a University of North Carolina evidence map of environmental health services in low-income and middle-income country health-care facilities, which searched PubMed, Scopus, and Global Health and was supplemented by hand-searching. Hand-hygiene-specific records were restricted to peer-reviewed English-language studies published between Jan 1, 2015, and Nov 28, 2025; backward citation chasing identified additional eligible records. Random-effects meta-analysis was done for studies with extractable pre-intervention and post-intervention hand hygiene compliance data. Other outcomes were synthesized using effect-direction methods. Barriers and enablers were synthesized thematically. The protocol was registered with PROSPERO, CRD420251252831.

**Findings:**

We screened 656 records and included 57 studies from 32 low-income and middle-income countries. Fifteen studies contributed to meta-analysis. Hand hygiene interventions were associated with higher post-intervention than pre-intervention compliance (pooled risk ratio 1·45, 95% CI 1·20–1·74), with substantial heterogeneity (I²=98%). Most studies used non-randomized designs. Multimodal WHO-style strategies and system-change interventions focused on alcohol-based hand rub availability, placement, or related infrastructure were the most common intervention categories. Effect-direction synthesis suggested favorable effects for compliance and system-related outcomes, whereas evidence for reductions in health-care-associated infections was less consistent. Common barriers included supply shortages, weak audit and data systems, staffing and workload pressures, and WASH infrastructure constraints; common enablers included training, monitoring and feedback, leadership support, reliable supplies, and implementation support.

**Conclusion:**

Hand hygiene interventions can improve observed compliance in low-income and middle-income country health-care facilities, but sustained gains depend on system supports that make hand hygiene feasible at the point of care. Programs should pair training and behavior-change strategies with reliable supplies, functional WASH infrastructure, audit-and-feedback routines, leadership accountability, and protected implementation time.

## Full Text Availability

The license terms selected by the author(s) for this preprint version do not permit archiving in PMC. The full text is available from the preprint server.

